# Irradiation dose response under hypoxia for the application of the sterile insect technique in *Drosophila suzukii*

**DOI:** 10.1371/journal.pone.0226582

**Published:** 2019-12-31

**Authors:** Fabiana Sassù, Katerina Nikolouli, Rui Pereira, Marc J. B. Vreysen, Christian Stauffer, Carlos Cáceres

**Affiliations:** 1 Department of Forest and Soil Sciences, Boku, University of Natural Resources and Life Sciences, Vienna, Austria; 2 Division of Nuclear Techniques in Food and Agriculture, Insect Pest Control Subprogramme, Joint FAO/IAEA, Vienna, Austria; University of Thessaly School of Agricultural Sciences, GREECE

## Abstract

Treating insects with a lower oxygen atmosphere before and during exposure to radiation can mitigate some of the negative physiological effects due to the irradiation. The irradiation of pupae under oxygen-reduced environment such as hypoxia or anoxia is routinely used in the sterile insect technique (SIT) of some tephritid species as it provides radiological protection. This treatment allows to have the sterile pupae already in sealed containers facilitating the shipment. SIT is an environment friendly control tactic that could be used to manage populations of *Drosophila suzuk*ii in confined areas such as greenhouses. The objectives of this study were to assess the effect of irradiation on the reproductive sterility in *D*. *suzuk*ii males and females under low-oxygen atmosphere (hypoxia) and atmosphere conditions (normoxia). Additionally, we assessed the differences in radiological sensitivity of pupae treated under hypoxia and normoxia conditions. Finally, the effect on emergence rate and flight ability of the irradiated *D*. *suzuk*ii adults exposed to doses that induced >99% of sterility were assessed. Pupae needed a 220 Gy irradiation dose to achieve >99% of egg hatch sterility in males irrespective of the atmosphere condition. For females the same level of sterility was achieved already at 75 Gy and 90 Gy for the normoxia and hypoxia treatments, respectively. Radiation exposure at 170 and 220 Gy under the two atmosphere treatments did not have any effect on the emergence rate and flight ability of *D*. *suzukii* males and females. Therefore, hypoxia conditions can be used as part of an area-wide insect pest management program applying SIT to facilitate the protocols of packing, irradiation and shipment of sterile *D*. *suzukii* pupae.

## Introduction

The spotted wing Drosophila (SWD), *Drosophila suzukii* (Matsumura, 1931) (Diptera: Drosophilidae), is an invasive and polyphagous pest of Eastern Asian origin attacking several commercial soft-skinned fruits and berries in Europe and the Americas [1,2]. The female is equipped with a long-serrated ovipositor that can penetrate the epicarp of ripe soft-skinned fruits/berries [3]. The damage is caused by the feeding of the larvae on the fruit flesh and subsequently by infecting microorganisms resulting in reduced fruit quality [4,5] and economic losses [6–11].

The host fruits are infested close to the harvesting time making the control with broad-spectrum insecticides difficult [12]. Therefore, several approaches have been proposed to manage *D*. *suzukii* infestations like biological control tactics [13], cultural control practices [14], use of biorational pesticides [11], and mating disruption [15]. However, most of them appear to be partly effective and/or unsustainable [11,16,17].

Recently, the sterile insect technique (SIT) has been suggested as a potential complementary control method against *D*. *suzukii* [16,18–20]. The SIT is an environmentally friendly method based on the sequential, inundative releases of sterile insects in a targeted area. The released sterile males compete with wild males to mate with wild females [21]. A mating of a sterile male with a virgin wild female results in the production of non-fertile eggs and the reduction of offspring results in suppression or even, in specific situations, the local eradication of the wild population [22]. One of the key issues for the SIT application is to determine the radiation dose since the doses used to induce reproductive sterility may vary between sexes and among species [23,24]. As a sex separation system for *D*. *suzukii* is not available yet, both males and females need to be released in an SIT programme. Thus, it is crucial to study the impact of irradiation on *D*. *suzukii* female fertility and male mating competitiveness.

One of the crucial requirements for the successful implementation of an SIT programme is the establishment of protocols that will allow mass-production, sterilization and shipment of insects without detrimental effects on their biological quality parameters including male competitiveness during mating and longevity [25–27]. The negative effects of the radiation increase with increasing doses [28], and therefore, exposure to high levels of radiation may influence the physiology and behavior traits of the species [29]. The quality-reducing effects of ionizing radiation on insects are associated with the production of deleterious free radicals that are formed by the ionization of the intracellular water [30]. Exposing insects to a lower oxygen atmosphere such as hypoxia (low oxygen condition) or anoxia (zero oxygen condition) before and during exposure to radiation can mitigate some of these negative effects [31–34]. The irradiation of pupae under hypoxia is routinely used in the SIT programmes of tephritid species e.g. *Ceratitis capitata* and *Anastrepha ludens*, not only because it provides radiological protection, but because it also allows to have the sterile pupae already in sealed containers (e.g. plastic bags) which facilitates the packing and shipment of pupae to the final release destination [35]. However, irradiation under hypoxia atmosphere reduces the sensitivity of insects to radiation exposure, thus higher doses are needed to produce comparable reproductive sterility than in normoxia conditions [36,37].

The objectives of this study were: 1) to assess the effects on the induced sterility of a long-range of different radiation doses for *D*. *suzukii* males and females; 2) to compare irradiation effects under normoxia and hypoxia when pupae are irradiated under these conditions; and 3) to determine the effects of irradiation under normoxia and hypoxia on adult emergence rate and flight ability of *D*. *suzukii*.

## Materials and methods

### *Drosophila suzukii* colony

All flies used in this study were obtained from a colony maintained at the Insect Pest Control Laboratory, Joint FAO/IAEA Division of Nuclear Techniques in Food and Agriculture, Seibersdorf, Austria. The colony was established in 2014 with pupae from the Agricultural Entomology Unit of the Edmund Mach Foundation in San Michele All’Adige, Trento Province, Italy. Flies were kept in cages and were provided with water and adult diet containing a mixture of sugar and hydrolysate enzymatic yeast (MP Biomedicals) in a 3:1 ratio [38]. Controlled environment conditions of 22 ± 5°C, 65% ± 5% RH and a 14:10 (L: D) photoperiod were maintained. Artificial diet (brewer yeast 5%, sugar 11.15%, carrot powder 13.70%, sodium benzoate 0.19%, nipagin 0.15%, water 69.81%) served as egg-laying substrate and larval diet. The diet was changed daily and kept in the laboratory until larval development and pupation had been completed. After 11 days, pupae were separated from the diet and kept under the regular laboratory conditions.

### Pre-irradiation treatments

One day before adult emergence, two groups of 2000 pupae each were treated under normoxia or hypoxia, respectively. At this age, the pupae are dark brown with visible red eyes and wings and they are about 24 hours before emergence. The pupae of the normoxia group were maintained under normal laboratory conditions before irradiation, while the ones of the hypoxia group were sealed in a hermetic polyethylene bag for 5 hours at 18°C to allow them metabolize the oxygen present in the bag. To assess O_2_ depletion, a gas-sensor device (CheckMate3, Dansesor A/S, Ringsted, Denmark) was used to measure the oxygen level inside the bags. At the end of the hypoxia treatment, the concentration of oxygen was approximately 0.3% compared to 20.9% in the normoxia group. Both groups were irradiated the same day immediately after the end of the normoxia and hypoxia treatments.

### Irradiation treatment

Pupae of the hypoxia and normoxia treatments were kept in sealed and perforated polyethylene bags (10 x 7 cm), respectively, and exposed to gamma rays using a ^60^Co irradiator (Gamma Cell-220, Nordion, Canada). To ensure that the pupae received always the same dose, the bags where placed in the middle of the chamber by using a polystyrene cylinder as a support. Three 10 by 10 mm Gafchromic®HD-V2 dosimetry films (International Specialty Products, NJ, USA) were irradiated together with the pupae of each treatment to confirm the irradiation dose. The optical density of the films was measured 24 hours after the treatment using a Radiochromic reader (FWT-92D, Far West Technology, Inc., Goleta, CA, USA) [39]. The following irradiation doses were applied: 30, 50, 70, 90, 110, 130, 150, 170, 190, 210, 220, 230 and 240 Gy.

The irradiated pupae were placed in a cage until adult emergence. Only adults that emerged within 24 hours after the irradiation were used for the experiments while those that emerged later were discarded. The emerged flies were sexed under a short period of CO_2_ anesthesia. The effect of the irradiation on sterility was tested for each radiation dose on two types of crosses: 25 irradiated females x 25 non-irradiated males and 25 non-irradiated females x 25 irradiated males. Three additional doses (75, 80, 85 Gy) were tested on cross: irradiated females x non-irradiated males to determine the lowest dose able to confer complete sterility under hypoxia and normoxia treatments All doses were tested using pupae from different generations. For each dose a control cross (0 Gy) of 25 non-irradiated females x 25 non-irradiated males was performed. All crosses were replicated five times per dose. More details of the described protocol at: http://dx.doi.org/10.17504/protocols.io.76whrfe.

### Fecundity, fertility and reproduction index (RI)

Males and females from each cross were transferred to a cage (17 x 8 x 11.5 cm) and kept together for four days to ensure sexual maturation and complete insemination of all females. Adults were provided with water and diet *ad libitum*. On the fifth day, three fresh blueberries were placed on top of each cage to serve as oviposition sites. The blueberries were daily replaced, and eggs were carefully collected using forceps. The eggs were placed on a wet black net in a Petri dish filled with larval diet and counted. The collections were continued until 500 eggs had been obtained per replicate, per cross and per irradiation dose or for a maximum of three days. The hatching rate was recorded 48 hours after egg collection. All the experiments were carried out under the standard laboratory conditions.

Fecundity was assessed for the irradiated females x non-irradiated males cross and calculated as the total number of eggs collected per cage divided by the average number of eggs oviposited by the control cross. The hatching rate was assessed for all cross types. The Reproduction Index (RI) was used to determine the total direct radiation effect on female reproduction. The RI was developed as an index to express the female reproduction by combining fecundity and egg hatch after the irradiation exposure. The RI is calculated as [(fecundity * egg hatch) / 100].

The effect of radiation dose and atmosphere treatments on F1 progeny was also assessed for all cross types by counting the number of pupae and adult emergence rate. The emerged flies were sexed, and the sex ratio was calculated as the proportion of male and female adults.

### Effect of irradiation dose on emergence rate and flight ability

For the quality control tests, pupae were irradiated either with a dose at 170 Gy or 220 Gy. Five replicates containing 25 pupae each were used for each atmosphere treatment and control (0 Gy). The experiment was repeated twice (hereafter: “blocks”). To ensure the proper age of the pupae at the time of irradiation, they were observed under a stereoscope and only the dark-colored ones with visible red eyes and wings were selected [40].

For the emergence rate and flight ability tests the pupae were irradiated with the same procedures described above and immediately placed inside a paper ring in a Petri dish and covered it with a black PVC cylinder (8.4 cm internal diameter and 10 cm height) for 48 hours. The PVC cylinder was coated internally with talcum powder to prevent the emerging adults from crawling out of the cylinder instead of flying. The emergence rate of these pupae was estimated by subtracting non-emerged pupae and partially emerged flies from the total number of emerged flies. Fully emerged flies with and without body deformities were separately scored. Flies found inside the cylinder, but without any deformities were considered not able to fly and thus recorded as “no fliers”. The index of flight ability was calculated as the number of emerged flies, subtracted by the number of “no fliers”, and divided by the number of emerged flies as described in the “Product Quality Control for Sterile Mass-Reared and Released Tephritid Fruit Flies” manual [41]. Flies outside the tubes were removed every hour to avoid re-entry in the cylinders. No food or water was provided during the experiments.

### Data analysis

All statistical analyses were carried out using R 3.6 [42]. All data were binomially distributed and analyzed using the generalized linear models. Fecundity rate was analyzed using a generalized linear model with Poisson distribution [43]. To assess differences between hypoxia and normoxia and between irradiated and non-irradiated flies, irradiation doses, atmosphere treatments, their interaction and replicates were modeled as a fixed effect. The quality control parameters (adult emergence and flight ability) were analyzed using a generalized linear mixed models (binomial family) where the irradiation doses, atmosphere treatments, and replicates were included as fixed effects and blocks as a random effect. Tukey’s HSD corrections were used for multiple comparisons. For all data significance was set at α *=* 0.05. The dose-response male and female fertility under normoxia and hypoxia atmospheres were corrected against the fertility of the control cages and transformed using the normal equivalent deviate (N.E.D.). In all cases, the mean ± SD is reported.

## Results

### Fecundity

#### Crosses between non-irradiated females and irradiated males

The average number of eggs laid by the fertile females of the untreated control groups (376.7 ±143.2) did not differ compared to the hypoxia groups in all irradiation doses (328.1 ±142.2) (z-value = -1.838, P = 0.157). However, there was a difference in the number of eggs laid by the females of normoxia crosses, and the untreated control group (303.2 ±153.4 eggs) (z-value = -2.949, P = 0.008). No difference was detected in fecundity between the hypoxia and normoxia atmosphere conditions (z-value = -1.133, P = 0.493), but there was an effect of the interaction (all comparisons P < 0.002, data pooled for all irradiation doses and atmosphere treatments).

#### Crosses between irradiated females and non-irradiated males

The irradiation exposure decreased the fecundity of females irradiated under hypoxia (*z*-value = -43.41, P < 0.0001) and normoxia (*z*-value = -46.23, P < 0.0001) compared to control females. At 30 Gy the fecundity of the irradiated females was 25.9% under hypoxia and 10.4% under normoxia compared to the control group. In all irradiation doses, there was difference in the fecundity between hypoxia and normoxia atmosphere conditions (*z*-value = -14.08, P < 0.0001). The interaction between atmosphere conditions and irradiation doses had an effect on fecundity of females at dose from 30 Gy to 70 Gy (P < 0.01, data pooled). At 90 Gy, only one egg was laid by the irradiated females treated under hypoxia and 3 eggs from the irradiated females treated under normoxia (0.05% and 0.19% of the fecundity of the respective control groups) (S2 Fig).

### Fertility

#### Crosses between non-irradiated females and irradiated males

**Fig 1:**
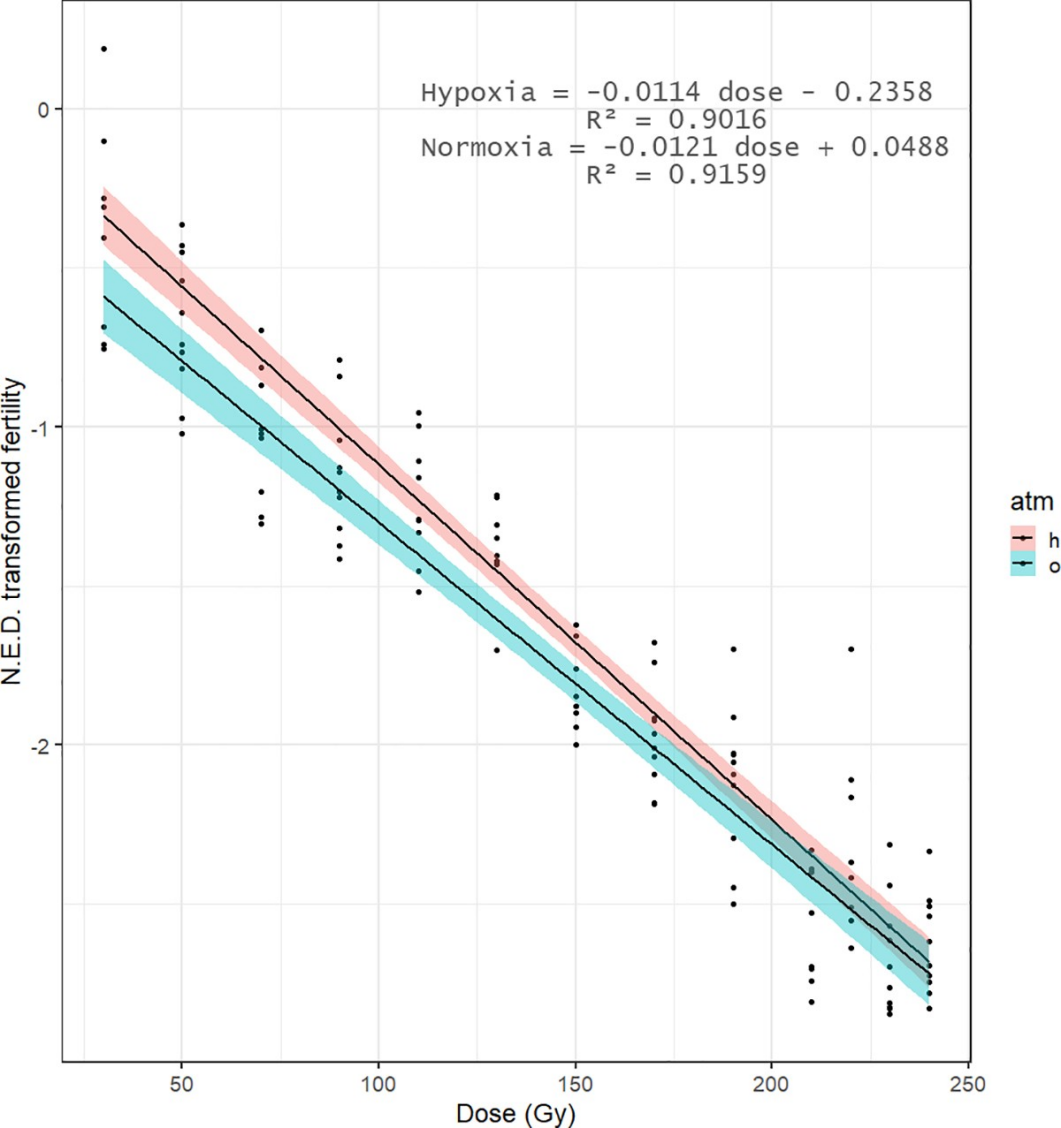
Linear regression of the transformed fertility of non-irradiated females and irradiated males. Linear regressions (bold lines) of the dose response fertility value (full dots) under hypoxia (”h”) and normoxia (”o”) atmosphere in a cross of irradiated males with non-irradiated females. The y-axis represents the irradiation doses (Gy); the x-axis represents the normal equivalent deviate transformation (N.E.D.) of the corrected fertility. Blue and red shaded areas represent the 95% confidence level interval for predictions from the linear model for the normoxia and hypoxia atmosphere, respectively.

The irradiation exposure had an effect on the male reproductive fertility compared to the control males (hypoxia: *z*-value = -13.921, P = 0.001; normoxia: *z*-value = -14.809, P = 0.001). In all irradiation doses, the reproductive fertility of irradiated males under hypoxia was different from that of the normoxia treated males (*z*-value = 2.443, P = 0.0387). On average, the dose necessary to obtain the same sterility level under hypoxia was 15 Gy higher than normoxia (Fig 1).

**Fig 2:**
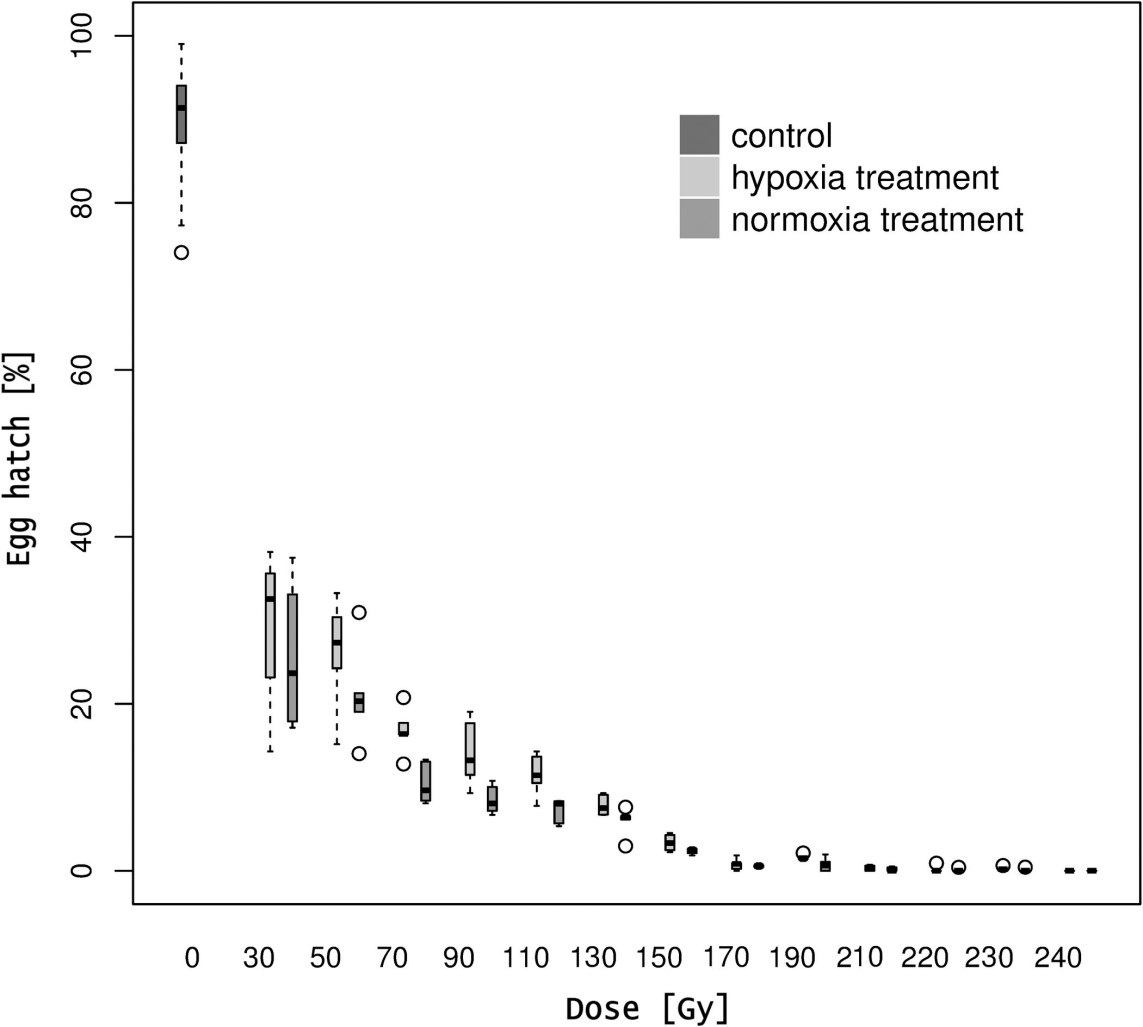
Percentage of egg hatch of non-irradiated females and irradiated males at different irradiation doses under hypoxia and normoxia atmosphere treatments. The effect of different irradiation doses on *D*. *suzukii* egg hatch under hypoxia and normoxia atmosphere conditions in a cross of irradiated males with non-irradiated females. Bold lines represent medians, dashed lines represent upper and lower whiskers and circles represent outliers.

Male irradiated under normoxia conditions reached more than 99% sterility levels when irradiated at 190 Gy while the same sterility level required 210 Gy under hypoxia conditions. Pupae irradiated at 220 Gy induced 99.8% of sterility under both normoxia and hypoxia conditions. A dose of 170 Gy induced 97% of sterility when males were treated under hypoxia (Fig 2). These two doses were selected to further study the effect of irradiation dose on the adult emergence and flight ability.

In all irradiation doses, the number of pupae and adults produced by the non-irradiated females and irradiated males cross was lower under hypoxia (pupae: *z*-value = 5.629, P < 0.0001; adult: *z*-value = 4.731, P < 0.0001) and normoxia (pupae: *z*-value = 5.483, P < 0.0001; adult: *z*-value = -4.524, P < 0.0001). There were not differences in the number of pupae and adults produced when irradiated males mated with non-irradiated females under hypoxia and normoxia (pupae: *z*-value = 0.437, P = 0.899; adult: *z*-value = 0.57, P = 0.568). At the lowest dose (30 Gy), the percentage of pupae and adults was less than 50% compared to the control regardless of the atmosphere treatment. Above 150 Gy, less than 1% of the F1 pupae completed the metamorphosis under either hypoxia or normoxia treatments. No F1 pupae were produced at 220 and 240 Gy under hypoxia and normoxia, although at 230 Gy there was a 0.06% and 0.09% pupae recovery under hypoxia and normoxia, respectively. No adults emerged when males were treated with 190 Gy under hypoxia and with 210 Gy under normoxia. Irradiation doses and atmosphere conditions did not affect the sex ratio of the emerged *F1* adults (hypoxia: F_1,90_ = 1.628, P = 0.20; normoxia: F_1,88_ = 0.5509, P = 0.46) (S1 Table).

#### Crosses between irradiated females and non-irradiated males

**Fig 3:**
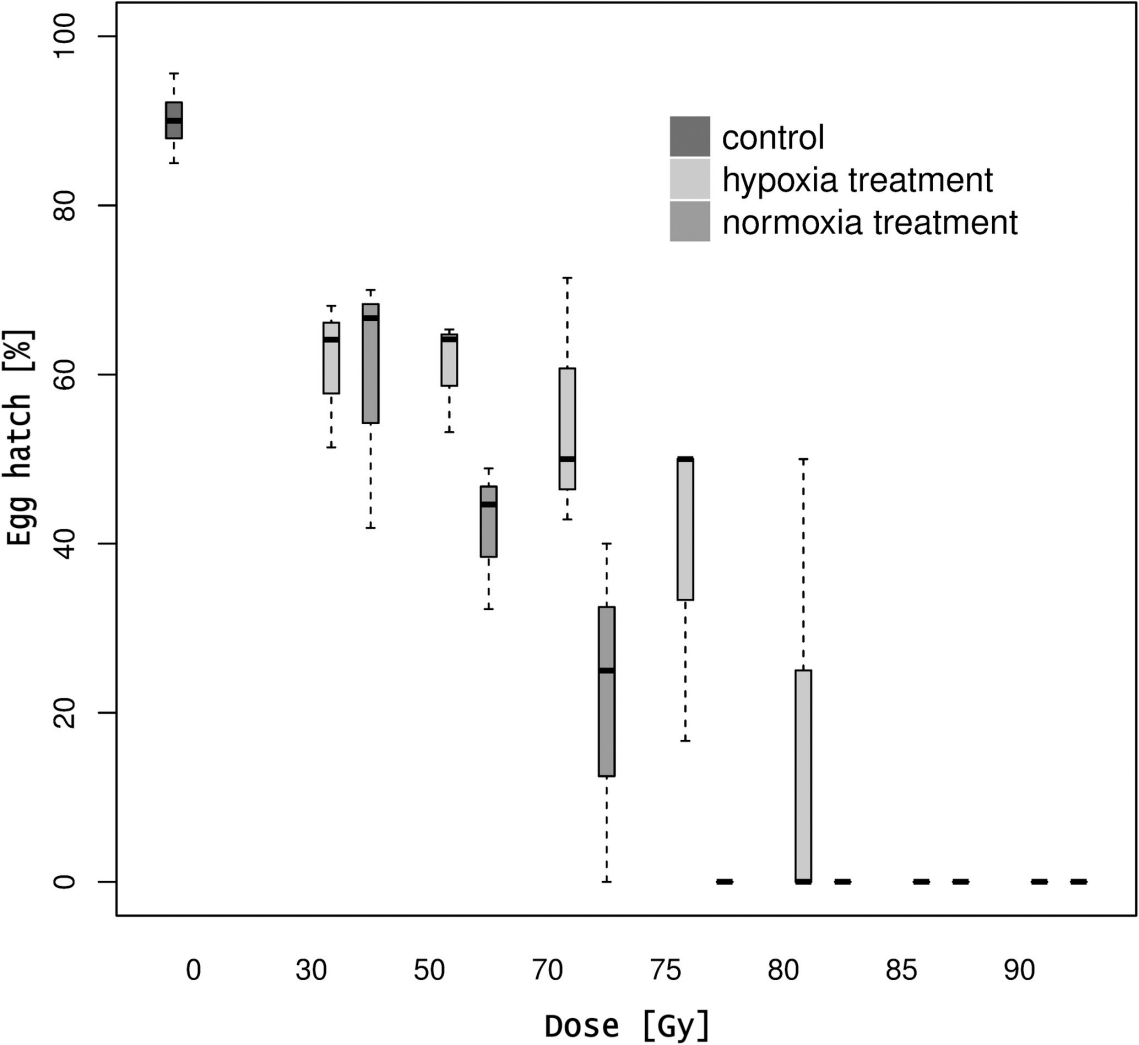
Percentage of egg hatch of irradiated females and non-irradiated males at different irradiation doses under hypoxia and normoxia atmosphere treatments. The effect of different irradiation doses on *D*. *suzukii* egg hatch under hypoxia and normoxia atmospheres in a cross of irradiated females with non- irradiated males. Bold lines represent medians and dashed lines represent upper and lower whiskers.

Irrespectively of the irradiation dose, fertility from irradiated females and non-irradiated males crosses was lower compared to the untreated control (hypoxia: z-value = 13.987, P < 0.001; normoxia: z-value = 14.267, P < 0.001). Except for the 30 Gy treatment group, fertility was higher under hypoxia conditions than normoxia (F_1,32_ = 7.3635, P = 0.01063). On average, the dose necessary to obtain the same sterility level under hypoxia was 10 Gy higher than normoxia (S1 Fig). Full sterility was obtained when females were treated at 75 and 85 Gy under normoxia and hypoxia conditions, respectively (Fig 3).

The females reproductive index (RI) at 70 Gy under normoxia was reduced to 0.12% compared to 90% of the control females. Similar data were obtained with females irradiated at 80 Gy under hypoxia conditions (0.13%) (S2 Table).

Females irradiated at 75 and 85 Gy under normoxia or hypoxia produced either no pupae or there was a failure in adult emergence. Atmosphere conditions did not affect the sex ratio of the emerged F1 adults (hypoxia: F_1,24_ = 0.347, P = 0.56; normoxia: F_1,19_ = 1.906, P = 0.18).

### Effect of irradiation dose on emergence rate and flight ability

**Fig 4:**
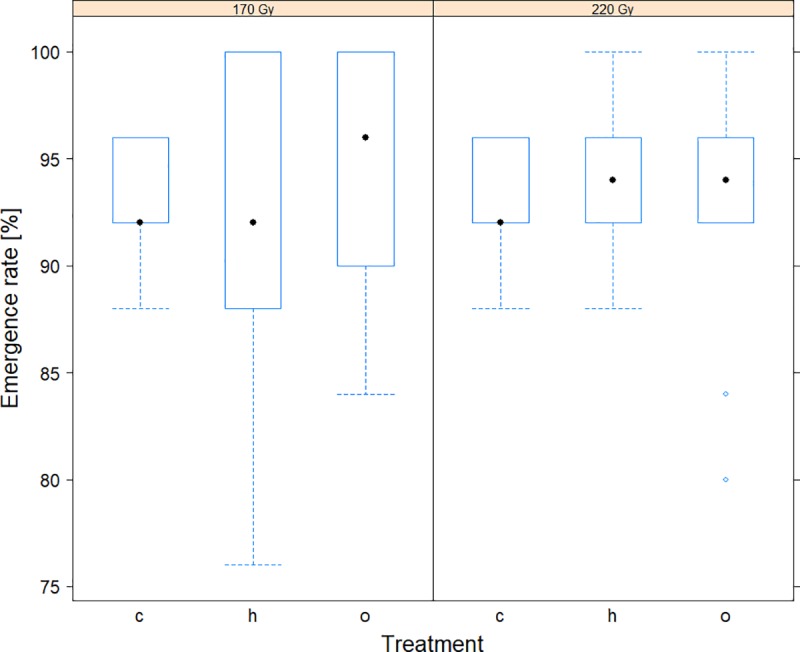
The effect of irradiation dose on the emergence rate. The effect of irradiation dose on the emergence rate of non-irradiated (“c”) and irradiated pupae at 170 and 220 Gy under hypoxia (“h”) and normoxia (“o”) conditions. Full dots represent medians, dashed lines represent upper and lower whiskers and circles represent outliers.

**Fig 5:**
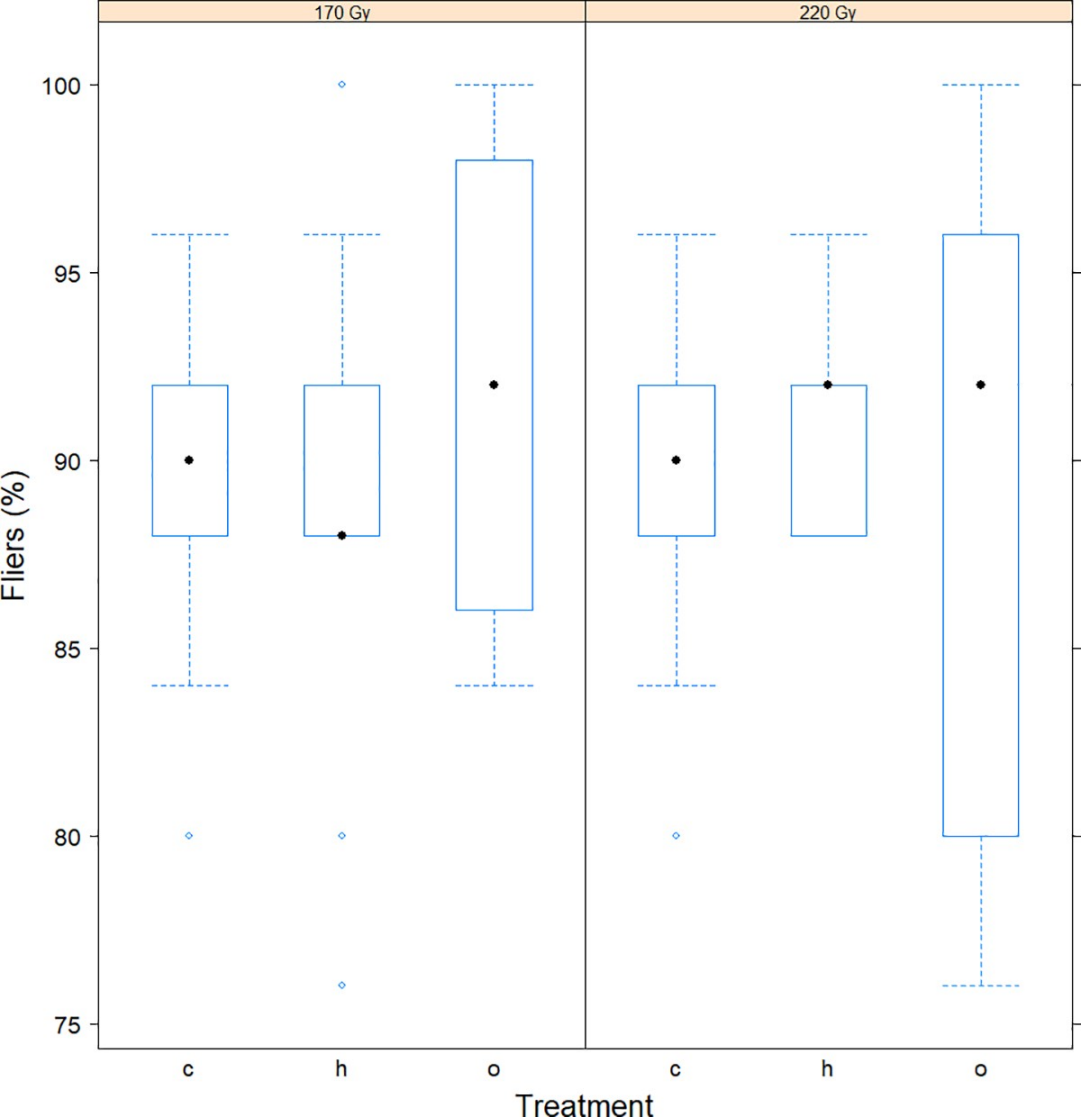
The effect of irradiation dose on the flight ability. The effect of irradiation dose on the flight ability rate of non-irradiated (“c”) and irradiated pupae at 170 and 220 Gy under hypoxia (“h”) and normoxia (“o”) conditions. Full dots represent medians, dashed lines represent upper and lower whiskers and circles represent outliers.

Male and female emergence rate and flight ability were assessed for pupae irradiated under normoxia or hypoxia at 170 Gy allowing a residual fertility of >3% [44] or at 220 Gy which induced a sterility of >99%. Emergence rate was independent of dose (*z*-value = 0.576, P *=* 0.565), atmosphere condition (*z*-value = 0.225, P = 0.972), and irradiation exposure (hypoxia: *z*-value = 0.221, P = 0.973; normoxia: *z*-value = 0.446, P = 0. 896). Adult emergence rate was 92.0% and 94.4% for the dose of 170 Gy, while 93.6% and 92.8% for the dose of 220 Gy under hypoxia and normoxia, respectively (Fig 4 and S3 Table). Flight ability was likewise independent of dose (*z*-value = 1.852, P = 0.064), atmosphere condition (*z*-value = 1.263, P *=* 0.416), and irradiation exposure (hypoxia: *z*-value = -0.220, P = 0.974; normoxia: *z*-value = 1.045, P = 0.548). Flight ability rate was 88.8% and 91.6% at 170 Gy, and 91.6% and 88.4% at 220 Gy under hypoxia and normoxia, respectively (Fig 5 and S3 Table).

## Discussion

In this study, a wide range of gamma radiation doses was tested on *D*. *suzukii* pupae to determine the reproductive sterility dose for the potential application of SIT on this pest. Due to the radiological protection given by hypoxia atmosphere during irradiation treatment in several insect species [26,33], doses were assessed under normoxia and hypoxia atmosphere treatments for *D*. *suzukii* males and females.

In both atmosphere conditions, sterility increased with the increase of the irradiation doses as already described for other insects [36,45]. Full male sterility was achieved when pupae were exposed to 190 and 210 Gy in normoxia and hypoxia, respectively. This confirmed that *D*. *suzukii* pupae are more radiological resistant under both atmosphere conditions than tephritid species which can be sterilized between a dose range of 70–120 Gy [46–51] and *D*. *melanogaster* that requires 160 Gy [52].

As currently, no sexing system is available for *D*. *suzukii*, both sexes will be released in the field in future SIT applications. In irradiated females and non-irradiated males crosses, the fecundity and fertility and consequently the reproductive index decreased as the doses increased. A 75 Gy dose was sufficient to obtain complete female sterility under normoxia, confirming previous studies [18,20]. However, a higher dose of 85 Gy was required to obtain the same full female sterility under hypoxia treatment. In both atmospheres, females required much lower doses than males to achieve the same sterility confirming that females are more radiologically sensitive compared to males [45, 32, 33].

We determined a suitable range of radiation doses that will allow choosing the dose and the sterility level to optimize the efficiency of SIT. Our results suggest that a dose of 220 Gy induces 99.8% sterility (based on egg hatch data) in males and it would be recommended in an eradication strategy while a lower dose of 170 Gy (97% sterility based on egg hatch data) would be recommended for suppression programmes. In any of those two-choice scenarios for SIT application, irradiated females will be fully sterile.

The assessment of hypoxia conditions as mentioned above is of crucial importance for the implementation of SIT against *D*. *suzukii* since low oxygen levels reduce the negative effects on cells caused by free radicals generated from ionizing radiation [53]. These highly reactive molecules can cause irreversible intracellular alterations [31]. Therefore, in AW-IPM programs that include releases of sterile adults, pupae have to be kept under hypoxia or anoxia conditions during the irradiation, facilitating also transportation to the final release center since hypoxia will also prevent emergence during shipment [54]. In our study, *D*. *suzukii* pupae were treated under hypoxia prior and during irradiation and were compared with pupae irradiated under normoxia. For the sterilization of *D*. *suzukii* males, higher doses of approximately 15 Gy were required to obtain the same level of sterility in individuals irradiated under hypoxia than under normoxia. This phenomenon has been previously reported for tephritid fruit flies [23,24,33,55], tsetse flies [56], and lepidoptera species such as *Cydia pomonella* [57] and *Trichoplusia ni* [58].

To determine the effect of irradiation doses under different atmospheric conditions on the quality of *D*. *suzukii*, emergence rate and flight ability were assessed. Our results suggest that the examined quality parameters were not influenced by exposure to radiation at either a dose of 170 Gy or 220 Gy. Despite the protective effects of the low-oxygen atmosphere, no differences were detected in the emergence rate or flight ability. Additional parameters should be tested to complement our understanding of the effect of both atmosphere and irradiation dose on the biology of *D*. *suzukii* males. These additional parameters must include mating studies under semi-field conditions to assess the competitiveness of irradiated males when in competition with fertile males for mating with wild females. Nevertheless, the development of a protocol to irradiate *D*. *suzukii* pupae under hypoxia is an important step toward the development of the SIT package for *D*. *suzukii* and its implementation in confined areas such as greenhouses.

## Supporting information

S1 Fig Linear regression of the transformed fertility of the irradiated females and non-irradiated males. Linear regressions (bold lines) of the dose response fertility values (full dots) under hypoxia (“h”) and normoxia (“o”) atmosphere in a cross of irradiated females with non-irradiated males. The y-axis represents the irradiation doses (Gy); the x-axis represents the normal equivalent deviate transformation (N.E.D.) of the corrected fertility. Blue and red shaded areas represent the 95% confidence level interval for predictions from the linear model for the normoxia and hypoxia atmosphere, respectively.
(TIF)

S2 Fig Fecundity of irradiated females and non-irradiated males at different irradiation doses under hypoxia and normoxia atmosphere treatments. The effect of different irradiation doses on *D*. *suzukii* fecundity under hypoxia and normoxia atmosphere conditions in a cross of irradiated females with non-irradiated males. Bold lines represent medians, dashed lines represent upper and lower whiskers.
(TIF)

S1 Table Data of non-irradiated females and irradiated males experiment. Fertility, pupae recovery, adult emergence and sex ratio in crosses between irradiated males under hypoxia (“h”) and normoxia (“n”) conditions and non-irradiated females. The averaged percentage +/- SD of all replicates at different irradiation doses is presented. The sex ratio is presented in proportion.
(PDF)

S2 Table Data of irradiated females and non-irradiated males experiment. Fecundity, fertility, pupae recovery, adult emergence and sex ratio in crosses between irradiated females under hypoxia (“h”) and normoxia (“n”) conditions and non-irradiated males. The averaged percentage +/- SD of all replicates at different irradiation doses is presented. The sex ratio is presented in proportion.
(PDF)

S3 Table Data on emergence rate and flight ability experiment. Emergence and flight ability tests. The averaged percentage +/- SD of all replicates at the irradiation doses of 170 and 220 Gy is presented.
(PDF)

S4 Table Raw-data of non-irradiated females and irradiated males experiment. The number of eggs laid, egg hatch, pupae recovery, adult emergence and male adults in crosses between irradiated males under hypoxia (“h”) and normoxia (“n”) conditions and non-irradiated females. The mean +/- SD of all replicates at different irradiation doses is presented.
(PDF)

S5 Table Raw-data of irradiated females and non-irradiated males experiment. The number of eggs laid, egg hatch, pupae recovery, adult emergence and male adults in crosses between irradiated females under hypoxia (“h”) and normoxia (“n”) conditions and non-irradiated males. The mean +/- SD of all replicates at different irradiation doses is presented.
(PDF)
