# Supplementary figures and images for: Irradiation dose response under hypoxia for the application of the sterile insect technique in Drosophila suzukii
Source: PLoS One. 2019 Dec 31;14(12):e0226582. doi: 10.1371/journal.pone.0226582 (PMC6938351; doi:10.1371/journal.pone.0226582)

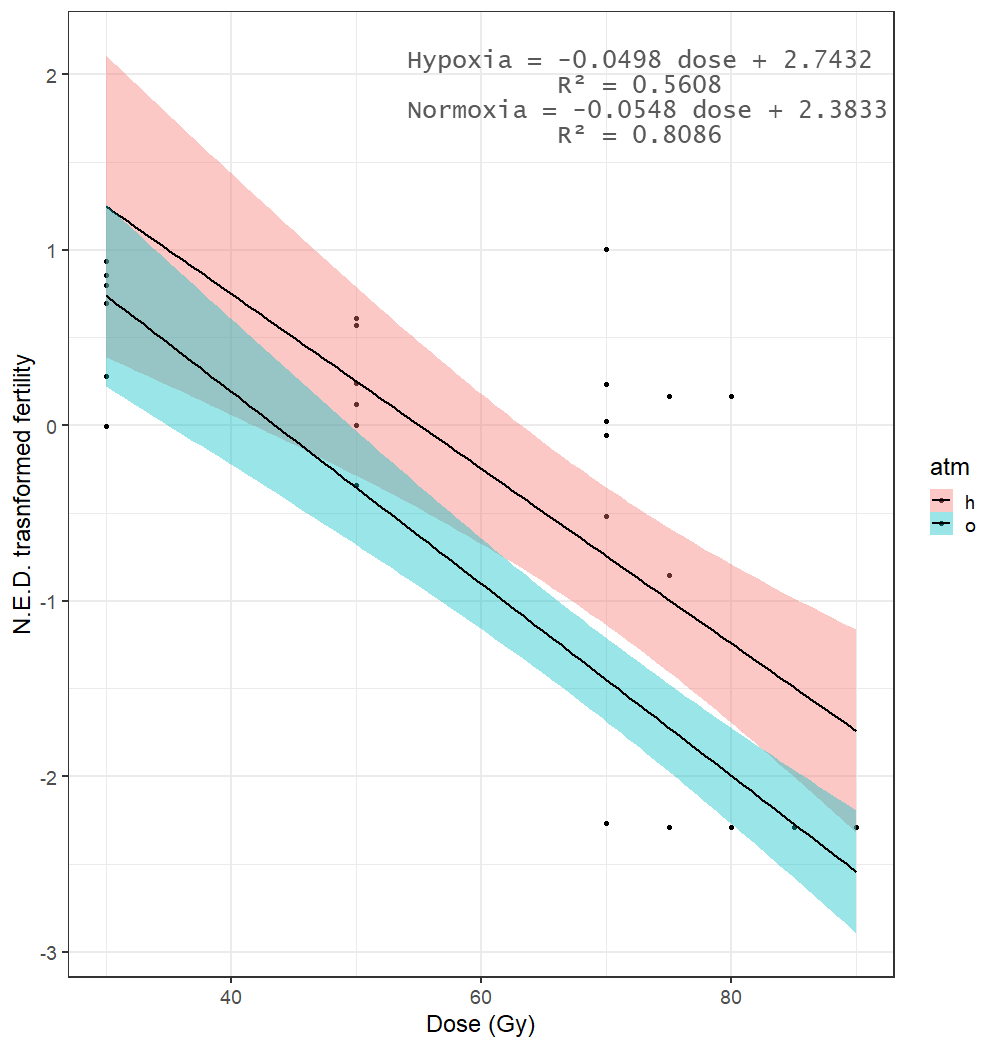

Supplement: S1 Fig — Linear regressions (bold lines) of the dose response fertility values (full dots) under hypoxia (“h”) and normoxia (“o”) atmosphere in a cross of irradiated females with non-irradiated males. The y-axis represents the irradiation doses (Gy); the x-axis represents the normal equivalent deviate transformation (N.E.D.) of the corrected fertility. Blue and red shaded areas represent the 95% confidence level interval for predictions from the linear model for the normoxia and hypoxia atmosphere, respectively. (TIF) [file pone.0226582.s001.tif]

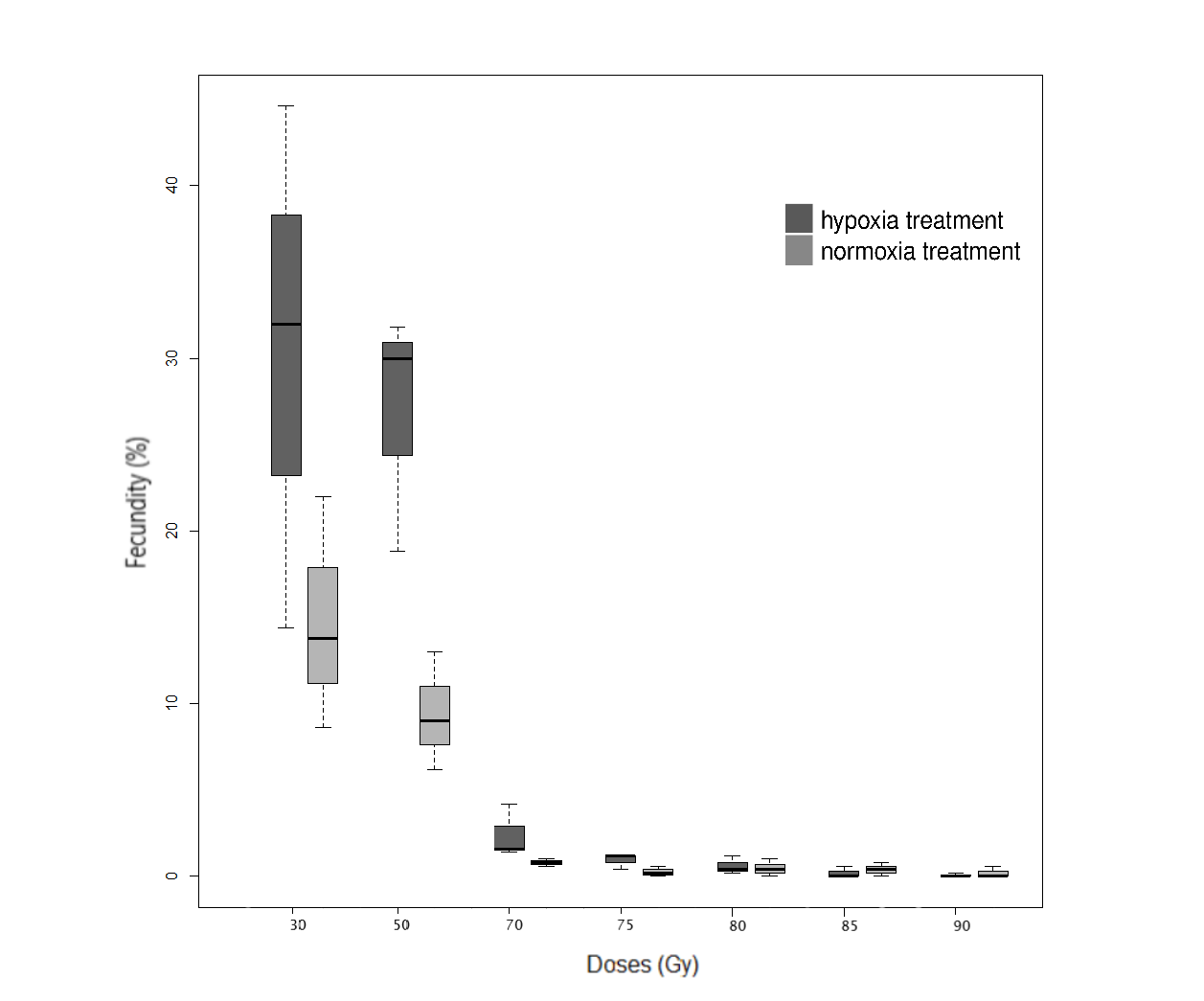

Supplement: S2 Fig — The effect of different irradiation doses on D. suzukii fecundity under hypoxia and normoxia atmosphere conditions in a cross of irradiated females with non-irradiated males. Bold lines represent medians, dashed lines represent upper and lower whiskers. (TIF) [file pone.0226582.s002.tif]
