# Supplementary material for: Irradiation dose response under hypoxia for the application of the sterile insect technique in Drosophila suzukii
Source: PLoS One. 2019 Dec 31;14(12):e0226582. doi: 10.1371/journal.pone.0226582 (PMC6938351; doi:10.1371/journal.pone.0226582)
Supplement: S1 Table — Fertility, pupae recovery, adult emergence and sex ratio in crosses between irradiated males under hypoxia (“h”) and normoxia (“n”) conditions and non-irradiated females. The averaged percentage +/- SD of all replicates at different irradiation doses is presented. The sex ratio is presented in proportion. (PDF) [file pone.0226582.s003.pdf]

Table S1 Data of non-irradiated females and irradiated males experiment.

Fertility, pupae recovery, adult emergence and sex ratio in crosses between irradiated males under hypoxia ("h") and normoxia ("n") conditions and non-irradiated females. The averaged percentage +/- SD of all replicates at different irradiation doses is presented. The sex ratio is presented in proportion.

	Fertility (%)		Pupae recovery (%)		Adult emergence (%)		Sex ratio (♀/♂)	
Dose	h	n	h	n	h	n	h	n
0 Gy	90.21 +/- 4.66		70.04 +/- 10.85		61.44 +/-		1:1	
30 Gy	29.39 +/- 10.4	25.50 +/- 8.23	22.54 +/- 13.69	16.63 +/- 8.18	13.94 +/- 9.0	13.24 +/- 8.0	1:1	1:1
50 Gy	26.07 +/- 6.96	21.12 +/- 6.15	21.61 +/- 32.88	14.78 +/- 22.98	20.42 +/- 34.23	5.57 +/- 15.46	1:1	2:1
70 Gy	16.80 +/- 2.86	9.96 +/- 2.52	13.50 +/- 16.53	8.65 +/- 16.68	12.18 +/- 14.74	8.02 +/- 12.65	1:1	1:1
90 Gy	14.15 +/- 4.12	8.55 +/- 1.78	8.88 +/- 8.45	6.16 +/- 8.23	8.37 +/- 7.66	5.44 +/- 6.02	1:2	1:2
110 Gy	11.53 +/- 2.16	7.15 +/- 1.50	2.12 +/- 3.27	1.88 +/- 10.42	1.05 +/- 2.30	1.37 +/- 7.5	2:1	1:1
130 Gy	7.90 +/- 1.25	5.90 +/- 1.75	1.72 +/- 1/41	1.02 +/- 3.34	1.21 +/- 0.83	0.67 +/- 3.03	1:1	0:1
150 Gy	3.37 +/- 1.04	2.38 +/- 0.3	0.48 +/- 1.79	0.46 +/- 1.65	0.36 +/- 0.89	0.30 +/- 1.51	1:1	0:1
170 Gy	2.98 +/- 0.71	1.86 +/- 0.24	0.93 +/- 3.56	0.78 +/-	0.76 +/- 3.67	0.57 +/-	1:1	1:3
190 Gy	1.57 +/- 0.35	0.73 +/- 0.82	0.12 +/- 0.54	0.06 +/- 0.44	0.00	0.06 +/- 0.44	-	1:1
210 Gy	0.32 +/- 0.33	0.19 +/- 0.25	0.09 +/- 0.54	0.08 +/- 0.44	0.00	0.00	-	-
220 Gy	0.16 +/-0.40	0.10 +/- 0.18	0.00	0.00	0.00	0.00	-	-
230 Gy	0.17 +/- 0.25	0.14 +/- 0.19	0.06 +/- 0.44	0.09 +/- 0.54	0.06 +/- 0.44	0.04 +/- 0.44	-	-
240 Gy	0.00	0.00	0.00	0.00	0.00	0.00	-	-
