# Supplementary material for: Irradiation dose response under hypoxia for the application of the sterile insect technique in Drosophila suzukii
Source: PLoS One. 2019 Dec 31;14(12):e0226582. doi: 10.1371/journal.pone.0226582 (PMC6938351; doi:10.1371/journal.pone.0226582)
Supplement: S2 Table — Fecundity, fertility, pupae recovery, adult emergence and sex ratio in crosses between irradiated females under hypoxia (“h”) and normoxia (“n”) conditions and non-irradiated males. The averaged percentage +/- SD of all replicates at different irradiation doses is presented. The sex ratio is presented in proportion. (PDF) [file pone.0226582.s004.pdf]

Table S2 Data of irradiated females and non-irradiated males experiment.

Fecundity, fertility, pupae recovery, adult emergence and sex ratio in crosses between irradiated females under hypoxia (“h”) and normoxia (“n”) conditions and non-irradiated males. The averaged percentage +/- SD of all replicates at different irradiation doses is presented. The sex ratio is presented in proportion.

	% Fecundity		% Fertility		Reproductive Index		% Pupae recovery		% Adult emergence		Sex ratio (♀/♂)	
Dose	h	n	h	n	h	n	h	n	h	n	h	n
0 Gy	100		90. 46 +/- 2.92		90. 46 +/- 2.92		70.04 +/-		61.44 +/-		1:1	
30 Gy	25.88 +/- 12.6	10.35 +/- 7.81	61.21+/- 7.14	59.51 +/- 12.55	15.84+/-	6.16 +/-	45.72 +/- 3.28	45.09 +/- 11.9	36.05 +/- 2.66	43.05 +/- 13.30	1:1	1:1
50 Gy	24.53 +/- 13.81	8.67 +/- 6.78	60.89+/- 5.47	41.92 +/- 7.05	13.69 +/-	3.95 +/-	56.89 +/- 3.55	39.41 +/- 5.20	51.07 +/- 7.9	37.83 +/- 6.48	1:1	1:1
70 Gy	2.52 +/- 0.81	0.56 +/- 0.45	54.76 +/- 12.14	21.67 +/- 16.50	1.30 +/-	0.12 +/-	45.24 +/- 12.14	21.67 +/- 16.50	40.48 +/- 8.91	21.67 +/- 16.50	1:1	1:1
75 Gy	1.54 +/- 0.99	0.65+/- 0.23	38.89 +/- 15.71	0.00	0.60 +/-	0.00	33.33 +/- 13.61	0.00	27.78 +/- 15.71	0.00	1:1	-
80 Gy	0.79 +/-	1.35 +/- 1.04	16.67 +/- 23.57	0.00	0.13	0.00	16.67 +/- 23.57	0.00	16.67 +/- 23.57	0.00	1:1	-
85 Gy	0.37 +/- 0.12	0.56 +/- 0.42	0.00	0.00	0.00	0.00	0.00	0.00	0.00	0.00	-	-
90 Gy	0.05 +/- 0.03	0.19 +/- 0.04	0.00	0.00	0.00	0.00	0.00	0.00	0.00	0.00	-	-
