# Supplementary material for: Irradiation dose response under hypoxia for the application of the sterile insect technique in Drosophila suzukii
Source: PLoS One. 2019 Dec 31;14(12):e0226582. doi: 10.1371/journal.pone.0226582 (PMC6938351; doi:10.1371/journal.pone.0226582)
Supplement: S3 Table — Emergence and flight ability tests. The averaged percentage +/- SD of all replicates at the irradiation doses of 170 and 220 Gy is presented. (PDF) [file pone.0226582.s005.pdf]

Table S3 Data on emergence rate and flight ability experiment.

Emergence and flight ability tests. The averaged percentage +/- SD of all replicates at the irradiation doses of 170 and 220 Gy is presented.

	Emergence rate (%)		Flight ability rate (%)	
Dose	Hypoxia	Normoxia	Hypoxia	Normoxia
0 Gy	92.8 +/- 0.031	92.8 +/- 0.031	89.6 +/- 0.050	89.6 +/- 0.050
170 Gy	92.0 +/- 0.075	94.4 +/- 0.060	88.8 +/- 0.070	91.6 +/- 0.069
220 Gy	93.6 +/- 0.038	92.8 +/- 0.064	91.6 +/- 0.029	88.4 +/- 0.085
