# Supplementary material for: Irradiation dose response under hypoxia for the application of the sterile insect technique in Drosophila suzukii
Source: PLoS One. 2019 Dec 31;14(12):e0226582. doi: 10.1371/journal.pone.0226582 (PMC6938351; doi:10.1371/journal.pone.0226582)
Supplement: S4 Table — The number of eggs laid, egg hatch, pupae recovery, adult emergence and male adults in crosses between irradiated males under hypoxia (“h”) and normoxia (“n”) conditions and non-irradiated females. The mean +/- SD of all replicates at different irradiation doses is presented. (PDF) [file pone.0226582.s006.pdf]

Table S4 Raw-data of non-irradiated females and irradiated males experiment.

The number of eggs laid, egg hatch, pupae recovery, adult emergence and male adults in crosses between irradiated males under hypoxia (“h”) and normoxia (“n”) conditions and non-irradiated females. The mean +/- SD of all replicates at different irradiation doses is presented.

	Eggs laid		Eggs hatch		Pupae recovery		Adult emergence		Male	
Dose	h	n	h	n	h	n	h	n	h	n
0 Gy	371.7 +/- 143.2		335.3 +/- 131.0		238.1 +/-112.6		206.2 +/-101.2		102.43 +/-52.89	
30 Gy	301.0 +/- 102.7	385.7+/- 105.8	102.5 +/- 57.3	98.4 +/- 45.9	67.85+/- 37.2	64.15 +/- 45.9	41.9 +/- 27.6	51.0 +/- 38.3	25.1 +/- 4	27.7 +/- 5.3
50 Gy	421.6 +/- 82.9	297.4 +/- 91.3	113.2+/- 43.8	63.6 +/- 28.6	93.2+/- 32.8	44.8 +/- 22.9	88+/- 34.2	17.4 +/- 15.4	45.6 +/- 15.8	9.4 +/- 10.6
70 Gy	385 +/- 60.8	449.2 +/- 29.6	64.6 +/- 15.4	46.8 +/- 9.6	51 +/- 16.5	36 +/- 16.6	45.6 +/- 14.7	35.8 +/- 12.6	25.2 +/- 8.7	19 +/- 5.9
90 Gy	233.8 +/- 81.2	203.8 +/- 47.51	33 +/- 11.9	17.2 +/- 5.0	21 +/- 8.4	13.4 +/- 8.2	19.8 +/- 7.6	11.6 +/- 6.0	10.4 +/- 3.9	6.8 +/- 3.8
110 Gy	436.4 +/- 126.6	358.6 +/- 187.8	48.8 +/- 14.9	26.8 +/- 17.2	6.2 +/- 3.2	9.2 +/- 10.4	4.4 +/- 2.3	6.8 +/- 7.5	2.2 +/- 0.8	4.2 +/- 4.2
130 Gy	184.4 +/- 55.3	`172 +/- 83.4	14 +/- 2.7	10.6 +/- 6.2	3 +/- 1.4	2.4 +/- 3.3	2.2 +/- 0.8	1.8 +/- 3.0	1 +/- 1	1 +/- 2.3
150 Gy	421.8 +/- 93.1	478.4 +/- 99.2	13.6 +/- 2.8	11.6 +/- 3.7	2.2 +/- 1.7	2.2 +/- 1.6	1.6 +/- 0.8	1.4 +/- 1.5	0.8 +/- 0.8	0.8 +/- 1.3
170 Gy	395.8 +/- 157.1	469.6 +/- 63.8	9.4 +/- 6.6	9 +/- 5.5	3.8 +/- 3.5	3.8 +/- 2.0	3 +/- 3.6	2.8 +/- 1.4	1.6 +/- 2.0	1.4 +/- 1.1
190 Gy	341.8 +/- 85.2	179.6 +/- 93.7	5.4 +/- 1.9	1 +/- 1	0.4 +/- 0.5	0.2 +/- 0.4	0	0.2 +/- 0.4	0	0
210 Gy	373.6 +/- 82.0	336.8 +/- 65.4	1.4 +/- 1.51	0.6 +/- 0.8	0.4 +/- 0.5	0.2 +/- 0.4	0	0	0	0
220 Gy	126.2 +/- 98.9	150.5 +/- 94.4	0.25 +/- 0.5	0.25 +/- 0.5	0	0	0	0	0	0
230 Gy	420.6 +/- 69.0	432 +/- 70.2	0.6 +/- 0.8	0.6 +/- 0.8	0.2 +/- 0.4	0.4 +/- 0.5	0.2 +/- 0.4	0.2 +/- 0.4	0	0
240 Gy	327.2 +/- 161.1	269 +/- 92.6	0.4 +/- 0.8	0	0.4 +/- 0.8	0	0	0	0	0
