# Supplementary material for: Irradiation dose response under hypoxia for the application of the sterile insect technique in Drosophila suzukii
Source: PLoS One. 2019 Dec 31;14(12):e0226582. doi: 10.1371/journal.pone.0226582 (PMC6938351; doi:10.1371/journal.pone.0226582)
Supplement: S5 Table — The number of eggs laid, egg hatch, pupae recovery, adult emergence and male adults in crosses between irradiated females under hypoxia (“h”) and normoxia (“n”) conditions and non-irradiated males. The mean +/- SD of all replicates at different irradiation doses is presented. (PDF) [file pone.0226582.s007.pdf]

Table S5 Raw-data of irradiated females and non-irradiated males experiment.

The number of eggs laid, egg hatch, pupae recovery, adult emergence and male adults in crosses between irradiated females under hypoxia (“h”) and normoxia (“n”) conditions and non-irradiated males. The mean +/- SD of all replicates at different irradiation doses is presented.

	Eggs laid		Eggs hatch		Pupae recovery		Adult emergence		Male	
Dose	h	n	h	n	h	n	h	n	h	n
0 Gy	460.42 +/- 136.72		416 +/- 42		291.7 +/- 53		179.23 +/- 52		89.61 +/- 23	
30 Gy	151.67 +/- 61.93	74.0+/- 27.76	96.33 +/- 44.19	47.00 +/- 24.10	70.67+/- 32.71	36.33 +/- 22.69	54.00 +/- 22.09	35.33 +/- 23.43	28.00 +/- 11.61	21.67 +/- 17.00
50 Gy	134.33 +/- 28.76	47.0 +/- 13.95	83.33 +/- 23.63	20.33 +/- 7.85	77.33 +/- 20.04	18.33 +/- 7.04	68.33 +/- 17..17	18.33 +/- 7.04	39.33 +/- 3.86	7.33 +/- 4.11
70 Gy	12.00 +/- 6.38	4.00 +/- 0.82	7.33 +/- 5.44	1.00 +/- 0.82	6.00 +/- 4.32	1.00 +/- 0.82	5.0 +/- 2.94	1.00 +/- 0.82	2.00 +/- 0.82	0
75 Gy	4.67 +/- 1.89	1.33 +/- 1.25	1.67 +/- 0.82	0	1.00 +/- 0.82	0	1.00 +/- 0.82	0	0	0
80 Gy	3.00 +/- 2.16	2.33 +/- 2.05	0	0	0	0	0	0	0	0
85 Gy	1.0 +/- 1.41	2.00 +/- 1.63	0	0	0	0	0	0	0	0
90 Gy	0.33 +/- 0.47	1.00 +/- 1.41	0	0	0	0	0	0	0	0
